# Allergic Bronchopulmonary Aspergillosis (ABPA) With Colonized *Aspergillus fumigatus* Detected by Metagenomic Next‐Generation Sequencing on Tissue Samples: A Distinct Subset of ABPA With a Higher Risk of Exacerbation

**DOI:** 10.1111/crj.13794

**Published:** 2024-06-17

**Authors:** Wanjun Wang, Mo Xian, Yongxia Lei, Juhua Yang, Lulu Wu

**Affiliations:** ^1^ Department of Allergy and Clinical Immunology, Guangzhou Institute of Respiratory Health The First Affiliated Hospital of Guangzhou Medical University Guangzhou Guangdong China; ^2^ Department of Radiology The First Affiliated Hospital of Guangzhou Medical University Guangzhou Guangdong China; ^3^ Vision Medicals Co., Ltd Guangzhou China; ^4^ Department of Respiratory Medicine, Guangzhou Institute of Respiratory Health The First Affiliated Hospital of Guangzhou Medical University Guangzhou Guangdong China

**Keywords:** Allergic bronchopulmonary aspergillosis, asthma, metagenomic sequencing, colonization

To the Editor:

Allergic bronchopulmonary aspergillosis (ABPA) is an immunological pulmonary disorder caused by hypersensitivity to *Aspergillus*, which colonizes the airways of patients with asthma [1]. Previous studies have shown that fungal colonization in *Aspergillus*‐allergic diseases could lead to more rapid airway obstruction [2], but they lack a direct link to clinical relevance and outcome. Therefore, we aimed to identify *Aspergillus fumigatus* colonization in ABPA patients and compare their profile without colonization.

We retrospectively analyzed the clinical data of 116 patients at the Department of Allergy and Clinical Immunology, the First Affiliated Hospital of Guangzhou Medical University, from January 2014 to October 2020, of which 96 cases were diagnosed with ABPA according to the criteria described previously [3]. Twenty patients with the diagnosis of refractory asthma, according to the Global Institute for Asthma (GINA), were enrolled as controls. The definition of ABPA exacerbation was worsening of clinical status, obstructive deterioration of lung function, rising total IgE levels by > 50%, and the appearance of new shallow infiltrates on radiology. All patients underwent a trans‐bronchoscopy and bronchial mucosa biopsy. We utilized metagenomic next‐generation sequencing for the detection of the *A. fumigatus* genome from the samples. Details about the methods are in Appendix S1.

Our study showed that the basic demographic features were similar in subjects with or without tissue colonization, except for the expectoration of mucus plugs. Eosinophil counts; blood *A. fumigatus*‐sIgE, IgG, and total IgE levels; and the rate of *Pseudomonas aeruginosa* colonization were all higher in ABPA than in severe asthma. In subjects with colonization, an average of 690 ± 530 bp *A. fumigatus* nucleotide reads were detected, but only 3.9% had a positive sputum culture. The FEV_1_, FVC, and FEV_1_/FVC values were significantly lower in subjects with colonization. Higher bronchiectasis CT values (59.5 ± 7.1 vs. 34 ± 8, *p* < 0.05) and more numbers of involved lobes and segments were observed in subjects with colonization (4.8 ± 0.6 vs. 3.3 ± 1.2 and 16.2 ± 4.6 vs. 9.6 ± 4.9, both *p* < 0.05). The presence of HAM was seen in 76.4% of subjects with colonization. The duration of follow‐up among the three groups was nearly identical. The ABPA subjects received a higher dosage of ICS and a longer period of oral corticosteroids. More than half of the subjects with colonization (54.9%) were treated with itraconazole. A significantly larger proportion, 24/51 (47.1% vs. 24.4%), of subjects with colonization experienced ABPA exacerbations (Table 1). Twenty‐two of these 24 subjects experienced their first exacerbation after 12 months of the initial diagnosis (Appendix S2). The incidence rate of exacerbation was also significantly higher in subjects with colonization than those without colonization (216 vs. 132 per 1000 person‐years, respectively; *p* < 0.01). Subjects with ABPA had more corticosteroid side effects than severe asthmatic subjects (88.2% vs. 86.7% vs. 60%, *p* < 0.05) (Table 1).

**TABLE 1:** Baseline demographics, clinical features, treatment, and follow‐up details of the study ABPA patients.

	With tissue colonization	Without tissue colonization	Severe asthma
	*n* = 51	*n* = 45	*n* = 20
**Clinical feature**			
Age (years)	49 ± 14.4	45 ± 14.1	46 ± 13.9
Sex (M/F)	31/20	23/22	13/7
BMI (kg/m^2^)	23 ± 1.3	22 ± 1.5	23 ± 1.6
Duration of asthma (years)	9.6 ± 9.4	9.4 ± 9.7	9.3 ± 8.9
Nonsmokers, *n* (%)	45 (88.2)	39 (86.2)	18 (90.0)
Expectoration of mucus plugs, *n* (%)	21 (41.2)	8 (17.8)	3 (15)^α^
Chronic rhinosinusitis, *n* (%)	51 (100)	45 (100)	18 (90.0)
Misdiagnosis, *n* (%)	11 (21.6)	7 (15.6)	2 (10.0)
**Laboratory examination**			
Eosinophil count (10^9^/L)	1.43 ± 0.96	1.36 ± 0.97	0.98 ± 0.67^α′β′^
*A. fumigatus* sputum culture, *n* (%)	2 (3.9)	0	0
*A. fumigatus* nucleotides reads (bp)	690 ± 530	0	0
Serum *A. fumigatus*‐sIgE (kU/L)	21.8 ± 17.4	20.3 ± 18.2	0.09 ± 0.04^α′β′^
Serum total IgE (kU/L)	3040 ± 1436.3	2541.2 ± 1027.6	436 ± 98.5^α′β′^
Serum *A. fumigatus‐*IgG (AU/mL)	426.4 ± 129.2	389.3 ± 154.6	69.4 ± 25.8^α′β′^
*P. aeruginosa* colonization, *n* (%)	20 (39.2)	17 (37.8)	0 (0)^α′β′^
**Spirometry**			
FEV_1_ % predicted	52.0 ± 8.0	72.0 ± 11.2^α′^	73.6 ± 9.3^α′^
FVC % predicted	69.0 ± 12.3	76.8 ± 12.4^α′^	75.0 ± 11.5^α′^
FEV_1_/FVC ratio	50.0 ± 8.7	70.1 ± 14.1^α′^	71.5 ± 13.8^α′^
**HRCT findings**			
Bronchiectasis CT value	59.5 ± 7.1	34.0 ± 8.0^α^	‐
No. of lobes involved	4.8 ± 0.6	3.3 ± 1.2^α^	‐
No. of segments involved	16.2 ± 4.6	9.6 ± 4.9^α^	‐
Presence of HAM, *n* (%)	39 (76.4)	8 (17.8)^α′^	‐
**Treatment and follow‐up**			
Follow‐up duration, in months*	32 (16–68)	33 (16–69)	30 (15–63)
Dosage of ICS (BDPE), μg per day	690.8 ± 213.7	612.3 ± 205	596.9 ± 239^α′^
Oral glucocorticoids, in months*	16 (7–34)	15 (6–30)	10 (3–22)^α′β′^
Itraconazole therapy	28 (54.9)	12 (26.7)^α^	‐
Subjects with ABPA exacerbation	24 (47.1)	11 (24.4)^α^	‐
Number of exacerbations per 1000 person‐years	216	132^α′^	‐
**Prednisone side effects**			
Any side effect	45 (88.2)	39 (86.7)	12 (60.0)^αβ^
Weight gain	39 (76.5)	32 (71.1)	5 (30.0)^α′β′^
Cushingoid facies	32 (62.7)	29 (64.4)	4 (20.0)^α′β′^
Infections	5 (9.8)	3 (6.7)	0
Hypertension	16 (31.4)	17 (37.8)	1 (5.0)^αβ^
Hyperglycemia	13 (25.5)	10 (22.2)	2 (10.0)
Cataracts	7 (13.7)	6 (11.1)	1 (5.0)
Osteoporosis	22 (43.1)	13 (28.9)	5 (25.0)

*Note:* Number of exacerbations per 1000 person‐years = (sum of the number of exacerbations experienced by each participant/sum of the number of years each participant was followed up) × 1000.

Abbreviations: *A. fumigatus*, *Aspergillosis fumigatus*; BDPE, beclomethasone dipropionate equivalent; FEV_1_, forced expiratory volume in 1 s; FVC, forced vital capacity; HAM, high‐attenuation mucus; IgE, immunoglobulin E; *P. aeruginosa*, *Pseudomonas aeruginosa* .

^α^ *p* < 0.05 and ^α′^ *p* < 0.01 compared with patients with *A. fumigatus* colonization.

^β^ *p* < 0.05 and ^β′^ *p* < 0.01 compared with patients without *A. fumigatus* colonization.

* Median (interquartile range).

In the study, we observed that ABPA tissue colonization by *A. fumigatus* was associated with a significantly worse clinical disease status and a higher risk of exacerbations. This seemed inconsistent with the previous study [4]. The discrepancy might be partly attributed to the higher specificity of metagenomic sequencing to identify microbiota colonization than conventional approaches (e.g., sputum cultures or real‐time PCR) [5, 6]. Besides, not all patients could afford the cost of itraconazole in China, so most of them received prednisone as their initial treatment. Long‐term corticosteroid therapy would facilitate *A. fumigatus* colonization in the lower airways [7]. Therefore, targeted intervention against the antigens soon after metagenomic sequencing confirmation and long‐term antifungal therapy might be beneficial to reduce future exacerbation risks. However, up to 76% of patients had received one course of antifungal treatment with a poor response. Starting treatment with omalizumab or mepolizumab may have a role as a complementary therapeutic option with antifungals [8]. If effective, these biologics may positively impact hospitalization rates for ABPA exacerbations and reduce healthcare expenditures. In conclusion, our findings reminded clinicians to consider a diversity of personalized treatments according to the status of colonization.

## Conflicts of Interest

The authors have no conflict of interest to declare.

## Supporting information


**Appendix S1** Diagnosis of tissue colonization by *A. fumigatus* by means of metagenomic sequencing in ABPA.

**Appendix S2** Time to first ABPA exacerbation. Symbols indicate when an individual patient’s follow‐up ended without ABPA exacerbation.

## Data Availability

The data that support the findings of this study are available from the corresponding author upon reasonable request.
