# Supplementary material for: Allergic Bronchopulmonary Aspergillosis (ABPA) With Colonized Aspergillus fumigatus Detected by Metagenomic Next‐Generation Sequencing on Tissue Samples: A Distinct Subset of ABPA With a Higher Risk of Exacerbation
Source: Clin Respir J. 2024 Jun 17;18(6):e13794. doi: 10.1111/crj.13794 (PMC11182735; doi:10.1111/crj.13794)
Supplement: Supplementary file 1 — Appendix S1 Diagnosis of tissue colonization by A. fumigatus by means of metagenomic sequencing in ABPA. [file CRJ-18-e13794-s001.docx]

**Methods**

**Specimen collection**

After confirming no risk of bleeding [including platelets (PLT) ≥ 50×10^9^/L, activated partial thromboplastin time (APTT) ≤ 50 s, fibrinogen (FIB) ≥ 1.5 g/L)], bronchoscopy (routine bronchoscopy [BF-F260, 5.5-mm outer diameter of the tip (Olympus, Tokyo, Japan) and ultrathin bronchoscopy [BF-P-260F, 4-mm outer diameter of the tip (Olympus)]) were performed by the physicians of the Respiratory Endoscopy Center to obtain specimens including bronchial mucosa. 6–8 pieces of specimens from each patient were equally divided into two parts for metagenomic sequencing analysis and pathology examination respectively. Although enough biopsy samples were collected in most patients for both metagenomic sequencing and pathological detection, some patients only went through one sampling strategies for metagenomic sequencing due to long-term intolerance to bronchoscopy or difficulty in obtaining samples. The study was approved by the research ethics board of the First Affiliated hospital of Guangzhou Medical University.

**Specimen processing and metagenomic sequencing procedure**

Bronchial mucosal samples from ABPA patients were collected and cut into small pieces in accordance with standard clinical procedures in our hospital. A 1.5-mL microcentrifuge tube with 0.7 mL lysis buffer, tissue sample pieces, and 1 g of 0.5-mm glass bead were attached to a horizontal platform on a vortex mixer and agitated vigorously at 2800×g for 30 minutes. A 0.3-mL volume of precipitated sample was transferred to a new 1.5-mL microcentrifuge tube. DNA was extracted from samples using a QIAamp® UCP Pathogen DNA Kit (Qiagen) following the manufacturer’s instructions. Human DNA was removed using Benzonase (Qiagen) and Tween20 (Sigma)^1^. Libraries were constructed for the DNA and cDNA samples using a Nextera XT DNA Library Prep Kit (Illumina, San Diego, CA), and the quality was subsequently assessed by Qubit dsDNA HS Assay kit followed by High Sensitivity DNA kit (Agilent) on an Agilent 2100 Bioanalyzer. Library pools were then loaded onto an Illumina Nextseq CN500 sequencer for 75 cycles of single-end sequencing to generate approximately 20 million reads for each library. Trimmomatic^2^ was used to eliminate low-quality reads, duplicate reads, adapter contamination, and those shorter than 70 bp. Low-complexity reads were removed by Kcomplexity's default settings were used to eliminate low-complexity reads. By utilizing SNAP v1.0beta3.185 to match the human sequence data to the hg38 reference genome, the human sequence data were located and eliminated. The Kraken 2 criteria for choosing representative assemblies for microorganisms (bacteria, viruses, fungi, protozoa, and other multicellular eukaryotic pathogens) from the NCBI Assembly and Genome databases (https://benlangmead.github.io/aws-indexes/k2) were used to select pathogens and their genomes or assemblies for the creation of the microbial genome database Kcomplexity using default parameters. Microbial reads were aligned to the database using Burrows-Wheeler Aligner software. The reads with 90% identity of reference were defined as mapped reads. The clinical reportable range for pathogens was established according to a previous study^3^. A positive detection was reported for a given species or genus if the reads per million (RPM) ratio, or RPM-r was ≥5, where the RPM-r was defined as the RPM_sample_ / RPM_NC_ (i.e., the RPM corresponding to a given species or genus in the clinical sample divided by the RPM in the NC/negative control)^4^. In addition, to minimize cross-species misalignments among closely related microorganisms, we penalized (reduced) the RPM of microorganisms sharing a genus or family designation, if the species or genus appeared in non-template controls.


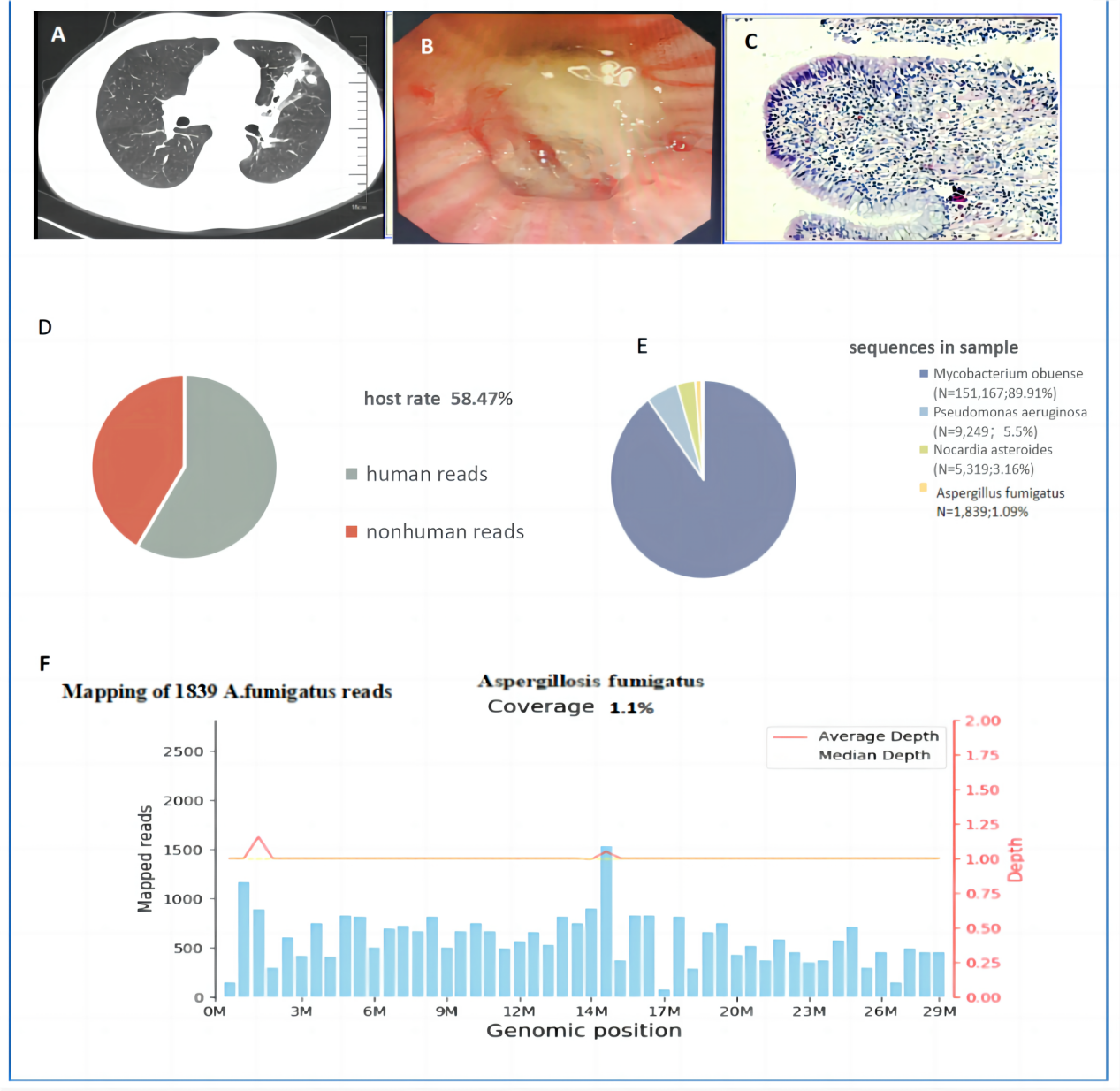


Appendix S1. Diagnosis of tissue colonization by *A.fumigatus* by means of metagenomic sequencing in ABPA

HRCT scanning revealed subsegmental bronchectasis filled with high-attenuation mucus, where mucosa was the site of biopsy (Panel A and B). Microscopic examination of the bronchial mucosa biopsy specimen showing goblet cell proliferation, submucosal edema, basal membrane thickening, lymphocyte, plasma cells and eosinophil infiltration (haematoxylin and eosin 100×)(Panel C).The distribution of nucleotides reads for component of host was 58.47%, and the bacterial sequences identified in the patient’s bronchial mucosa sample included Mycobacterium obuense, Pseudomonas aeruginosa, and Nocardia asteroides, which are considered to be nonpathogenic body flora.(Panel D and E). A total of 1839 sequence reads derived from the patient’s bronchial mucosa sample were mapped to the closest matched *A.fumigatus* genome with 1.1% coverage rate in the reference database. (Panel F).

**Statistics**

Statistical analyses were performed using SPSS (version 20.0; SPSS, Chicago, USA). Continuous variables were presented as mean±standard deviation. Different groups were compared using Mann-Whitney U test or ANOVA/Kruskal-Wallis test with Dunn post hoc testing. The Mann-Whitney U test was used to analyse variables that were not normally distributed, such as the pulmonary function parameter FVC, FEV_1_, FEV_1_/FVC, total IgE, sIgE ,CT value. Qualitative variables were compared using the chi-squared test, such as the number of clinical features, number of lobes or segments involved, number of itraconazole therapy and the prednisone side effects. The incidence rate of ABPA exacerbation (sum of the number of exacerbations in each subject/sum of the number of years each subject was followed up, multiplied by 1000) was computed for ABPA subjects with or without *A.fumigatus* tissue colonization. Kaplan-Meier analysis was applied to study the time until first ABPA exacerbation. P value less than 0.05 was considered statistically.

**References**

1. Amar Y, Lagkouvardos I, Silva RL, et al. Pre-digest of unprotected DNA by Benzonase improves the representation of living skin bacteria and efficiently depletes host DNA. Microbiome. 2021;26;9(1):123.

2. Xu Y, Kang L, Shen Z, et al. Dynamics of severe acute respiratory syndrome coronavirus 2 genome variants in the feces during convalescence. *J Genet Genomics* 2020; 47(10): 610-7.

3. Jing C, Chen H, Liang Y, et al. Clinical Evaluation of an Improved Metagenomic Next-Generation Sequencing Test for the Diagnosis of Bloodstream Infections. *Clin Chem* 2021; 67(8): 1133-43.

4. Miller S, Naccache SN, Samayoa E, et al. Laboratory validation of a clinical metagenomic sequencing assay for pathogen detection in cerebrospinal fluid. *Genome Res* 2019; 29(5): 831-42.
