# Supplementary material for: Allergic Bronchopulmonary Aspergillosis (ABPA) With Colonized Aspergillus fumigatus Detected by Metagenomic Next‐Generation Sequencing on Tissue Samples: A Distinct Subset of ABPA With a Higher Risk of Exacerbation
Source: Clin Respir J. 2024 Jun 17;18(6):e13794. doi: 10.1111/crj.13794 (PMC11182735; doi:10.1111/crj.13794)
Supplement: Supplementary file 2 — Appendix S2 Time to first ABPA exacerbation. Symbols indicate when an individual patient’s follow‐up ended without ABPA exacerbation. [file CRJ-18-e13794-s002.docx]

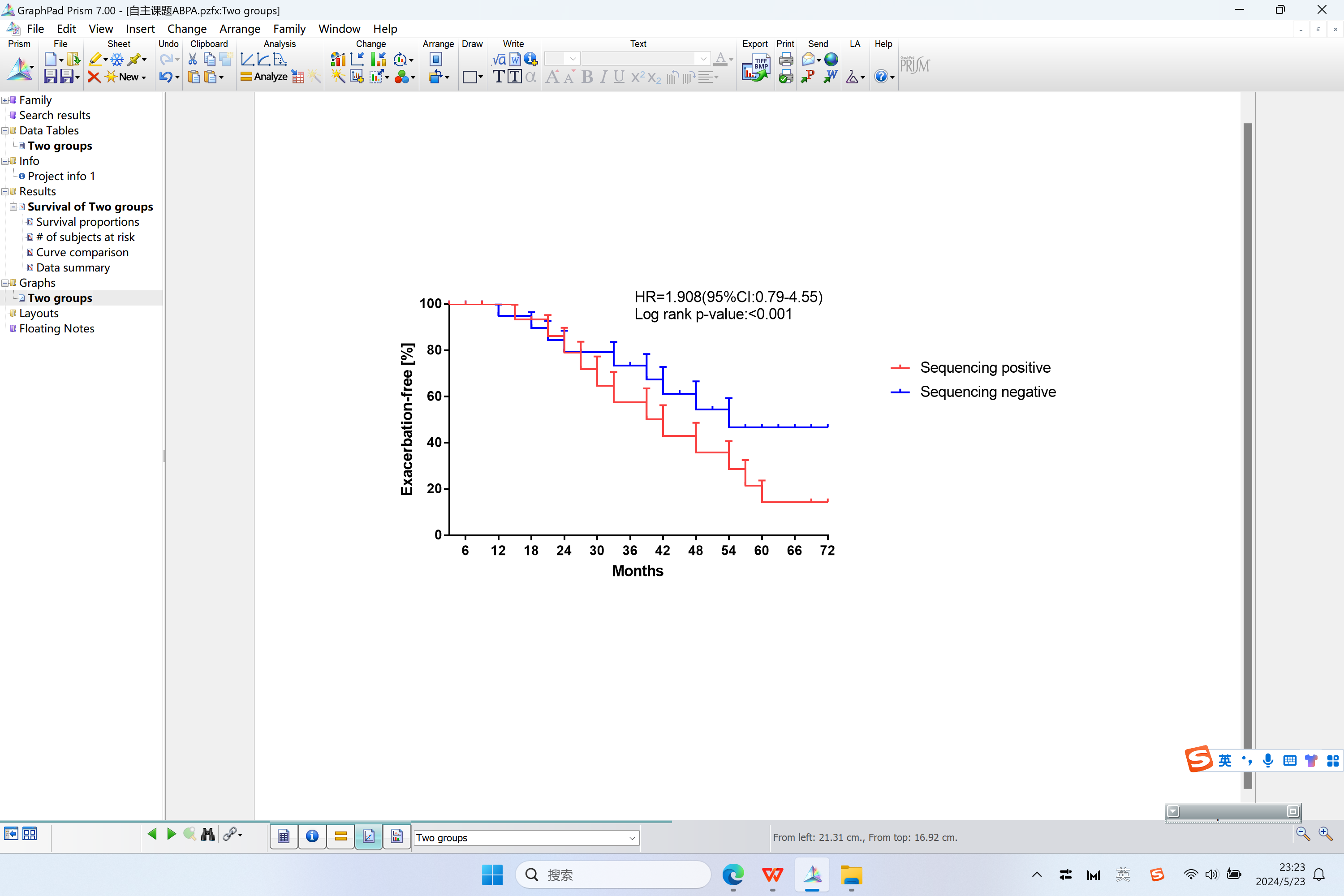


Subjects at risk, (n)

| *A.fumigatus* sequencing positive | 17 | 16 | 14 | 12 | 10 | 9 | 7 | 6 | 5 | 3 | 3 | 1 |
| --- | --- | --- | --- | --- | --- | --- | --- | --- | --- | --- | --- | --- |
| *A.fumigatus* sequencing negative | 22 | 20 | 18 | 16 | 15 | 13 | 11 | 9 | 7 | 5 | 3 | 1 |

Appendix S2. Time to first ABPA exacerbation. Symbols indicate when individual patient’s follow-up ended without ABPA exacerbation.
